# Proceedings of 2nd Annual Charles River World Congress: Lessons Learned from Rare Disease and Personalized Medicine Approaches

**DOI:** 10.1186/s12967-018-1596-2

**Published:** 2018-09-14

## A1 The Cystic Fibrosis Foundation’s venture philanthropy model

### Preston W. Campbell

#### Cystic Fibrosis Foundation, Bethesda, MD, USA

##### **Correspondence:** Preston W. Campbell

*Journal of Translational Medicine* 2018, **16(Suppl 2):**A1

The Cystic Fibrosis Foundation’s venture philanthropy model has been highly successful in bringing transformational therapies to people with CF. The CF Foundation established its care center network, which concentrated the patient population in centers of excellence and improved CF care, coupled with existing therapies, the care center network contributed to significant gains in survival and a CF patient registry was started at the same time. Together, these programs enabled clinical trials in adults with CF, with study design guided by registry epidemiological data. In 1998 began the first-ever discovery efforts to repair the dysfunctional CFTR. Three components of the program proved critical to its ultimate success: The CF Foundation offset companies’ risk by providing funding for discovery programs. CFTR assays were developed using human bronchial epithelial cells obtained from individuals with CF who had undergone lung transplant, and other scientific tools were readily shared. Academic scientists with relevant expertise were embedded in each industry program. In 2017, the CF Foundation met with 140 companies interested in entering the CF field and 50 clinical trials were conducted in its clinical trials network. Through its ongoing collaborative approach, the CF Foundation’s venture philanthropy program has brought hope to individuals with CF and their families.

## A2 Orphan to IND, the Jain Foundation’s efforts toward making a rare disease attractive to drug companies

### Doug Albrecht

#### Jain Foundation, Seattle, WA, USA

##### **Correspondence:** Doug Albrecht

*Journal of Translational Medicine* 2018, **16(Suppl 2):**A2

In 2005, the Jain Foundation was founded with the mission of finding a treatment for a member of the Jain family diagnosed with a rare genetic muscle wasting disease called Limb Girdle Muscular Dystrophy 2B/Miyoshi Myopathy (LGMD2B/MM). The rarity of LGMD2B/MM meant there were no known treatments, or even answers to most of the questions the Jain family had following their son’s initial diagnosis. Since then, the Jain Foundation has pursued its mission with a focus on repurposing approved therapeutics and decreasing the risk profile of LGMD2B/MM, to attract the attention of pharma companies. The Jain Foundation has focused its efforts on two primary areas: improving methods to assess the efficacy of candidate therapies through a better understanding of the disease process that occurs in LGMD2B/MM, and preparing the LGMD2B/MM patient community for participating in clinical trials. A retrospective analysis of the Jain Foundation’s programs that have led to a first therapeutic clinical trial for LGMD2B/MM will be covered.

## A3 Beyond clinical trials: reshaping drug development with expanded access and real-world data

### Martin Naley

#### myTomorrows, Boston, MA, USA

##### **Correspondence:** Martin Naley

*Journal of Translational Medicine* 2018, **16(Suppl 2):**A3

Sometimes a successful clinical trial just isn’t enough. As a company brings its newly-approved treatment to market, it faces questions from payers and doctors. Does the drug work in older patients, patients with poor kidney function, certain comorbidities, etc? It would be too risky to include those patients in the trial, so often these questions are left to be answered in post-market studies, which are costly and time-consuming. Another pathway exists, providing treatment access to patients in trial-adjacent populations and learning from them. This talk will describe a combined approach of expanded access and real-world data capture that can make treatment development and commercialization more efficient.

## A4 Is open science a viable option for discovering, developing and commercializing drugs for orphan diseases

### Owen Roberts

#### Meds4Kids Pharma, Toronto, Canada

##### **Correspondence:** Owen Roberts

*Journal of Translational Medicine* 2018, **16(Suppl 2):**A4

As we continue to expand our knowledge of the specific genetic mutations which cause many diseases, we have sub-divided what were once considered large unmet medical needs into smaller subsets amenable to precision medicines. However, as the patient populations of each of these subsets decrease, the cost of discovering, developing and commercializing the targeted medicines has remained the same. M4K Pharma, was created to address this problem by using an open science business model, which commits to, and encourages its partners, to share and publish their work and data without patents. In doing so, M4K intends to align and aggregate the work of granting institutions, academics, foundations and corporations in a cost-effective manner to discover, develop and register new therapeutics for orphan diseases.

## A5 The development of substrate reduction drugs for the treatment of glycosphingolipidoses

### James Shayman

#### University of Michigan, Ann Arbor, MI, USA

##### **Correspondence:** James Shayman

*Journal of Translational Medicine* 2018, **16(Suppl 2):**A5

Gaucher disease type 1 and Fabry disease represent the two most prevalent sphingolipid storage disorders. The standard for treatment of these diseases has been enzyme replacement therapy, limited by its expense, requirement for intra-venous infusion, loss of activity due to antibody formation, and lack of CNS penetrance. An alternative treatment strategy is the inhibition of glucosylceramide synthesis which blocks the accumulation of lysosomal glycosphingolipids. Early efforts in the pursuit of this strategy led to the identification of potent inhibitors of glucosylceramide synthase with limited off target effects. Eliglustat tartrate, the clinical lead that resulted from these efforts and was subsequently the basis for multiple phase 1, 2, and 3 trials leading to FDA approval in 2014 as the first stand-alone oral therapy for Gaucher disease type 1. However, eliglustat is unsuited for use in CNS associated glycosphingolipid storage disorders due to its inability to cross the blood brain barrier. Recent efforts have been focused on the design eliglustat analogues with good brain penetrance with the goal of developing the next generation of drugs that can be used for Gaucher disease type 3, the GM2 gangliosidoses that include Tay-Sachs and Sandhoff disease, and GM1 gangliosidosis.

## A6 Mighty voices: harnessing the power of patients and caregivers

### Mike Porath

#### TheMighty.com, Burbank, CA, USA

##### **Correspondence:** Mike Porath

*Journal of Translational Medicine* 2018, **16(Suppl 2):**A6

Patients and caregivers are driving more rare disease research than ever. Inspiring stories and insightful data from the world’s largest health community show the true impact people can have on each other by sharing their experiences. Learn the approaches researchers can use to better collaborate with the people whose lives they are trying to improve. Let’s help each other.

## A7 Update from 2017 award winners: The path to prevention of prion disease

### Sonia Vallabh, Eric Minikel

#### Prion Alliance, Broad Institute of MIT and Harvard, Cambridge, MA, USA

##### **Correspondence:** Sonia Vallabh, Eric Minikel

*Journal of Translational Medicine* 2018, **16(Suppl 2):**A7

Prion disease is an incurable and fatal neurodegenerative disease caused by misfolding of the prion protein (PrP). In individuals with mutations in the PrP gene, predictive genetic testing provides an opportunity to intervene years or decades before the onset of disease and preserve full quality of life, but therapeutic trials in this healthy population cannot rely on a conventional clinical endpoint. Instead, we will present a path to prevention trials centered around a surrogate biomarker endpoint informed by our clear understanding of the biological mechanism of prion disease.

## A8 AFMTelethon: a game changer patient organization in the world of medical research

### Serge Braun

#### AFM-Telethon, Evry, France

##### **Correspondence:** Serge Braun

*Journal of Translational Medicine* 2018, **16(Suppl 2):**A8

AFMTelethon has raised > $100 M/year for 29 years, which helped to reframe the whole field, bringing it from a rather remote scientific and commercially unattractive area to a very active and innovative ground. A striking demonstration is the comprehensive genetic map of the human genome published in 1992 and 1996 by its non-for-profit biotech Genethon; a quantum leap in the human genetics field. Not only patient organizations can serve as disease-specific experts, they more importantly (1) work on reducing bottlenecks/barriers for all levels of the process of product development, (2) initiate and drive research projects, (3) provide tools (e.g. databases, registries, large animal facilities, high-throughput screening, and even GMP biotherapeutics plateforms,…), and (5) represent a voice for all aspects (ethical, regulatory, technical) of innovative drug developments.

Through AFMTelethon initiatives, examples will be given of successful ecosystems, which also serve as models for frequent disorders. This includes shared scientific and financial risks in translational research by (1) directly funding and supporting drug development programs in industry or in academia (2) putting in place its own Research and Development as well as GMP biotherapeutic facilities, generating innovative drugs that are now either in the clinic or approved (3) founding start-up companies out of its own portfolio (4) taking equity shares in biotech companies, (5) launching a venture capital fund, and (6) incorporating a new type of private company dedicated to cell and gene therapy. The different actions presented are also driven by the growing challenge of fair price, which aims at ensuring patient access to the therapeutics.

## A9 Using a collaborative team approach to accelerate towards effective treatments for rare diseases

### Jill Weimer^1^, Antti Nurmi^2^

#### ^1^Sanford Health, Sioux Falls, SD, USA; ^2^Charles River, Wilmington, MA, USA

##### **Correspondence:** Jill Weimer

*Journal of Translational Medicine* 2018, **16(Suppl 2):**A9

CLN6-Batten disease is a progressive rare disease characterized by motor and cognitive impairments, seizures, and ultimately premature death. Pathologically, there is massive accumulation of autofluorescent storage material in lysosomes, gliosis and neurodegeneration. Over the last five years, our team has taken a team approach to move the needle towards having effective treatments for this fatal disorder, including development of a first-in-man gene therapy program for CLN6-Batten disease. In this presentation, we will discuss some of our successes as well as hurdles yet to be overcome. With the push towards more clinical trials for Batten disease patients, one consistent hurdle that has yet to be overcome is how to non-invasively monitor patient on trial for disease progression. Recently, our team has partnered with Charles Rivers to come up with a comprehensive biomarker scoring system in a mouse model of CLN6-Batten disease that combines imaging and behavior data to provide a biomarker scoring system for longitudinal disease monitoring. Identifying biomarkers associated with the multiple forms of Batten disease is an integral step towards the goal of improving early diagnosis, tracking progression of the disease, and monitoring response to treatment in both clinical and research environments.

## A10 Phenotypic screening as a source of novel molecules, targets and mechanisms in neurodegenerative disease

### Ken Rhodes

#### Yumanity Therapeutics, Cambridge, MA, USA

##### **Correspondence:** Ken Rhodes

*Journal of Translational Medicine* 2018, **16(Suppl 2):**A10

Phenotypic screening in living cells offers an approach to identification of new drug targets and molecules for treatment of neurodegenerative disease. Sophisticated chemical genetics analyses offer an approach to identifying the pathways and targets for molecules identified through phenotypic screening. Human patient derived neurons offer a substrate to determine that molecules identified in phenotypic screens modulate cellular processes that are relevant to neurodegenerative disease.

## A11 Human stem cell-derived adeno-associated viruses are highly efficient vectors for both gene transfer and nuclease-free gene editing

### Jeff Ellsworth

#### Homology Medicine, Bedford, MA, USA

##### **Correspondence:** Jeff Ellsworth

*Journal of Translational Medicine* 2018, **16(Suppl 2):**A11

Homology Medicines, Inc. is a genetic medicines company dedicated to transforming the lives of patients suffering from rare genetic diseases with significant unmet medical needs by curing the underlying cause of the disease. Homology’s proprietary platform is designed to utilize its human hematopoietic stem cell-derived adeno-associated virus vectors (AAVHSCs) to precisely and efficiently deliver genetic medicines in vivo either through a gene therapy or nuclease-free gene editing modality across a broad range of genetic disorders. Homology has a management team with a successful track record of discovering, developing and commercializing therapeutics with a particular focus on rare diseases, and intellectual property covering its suite of 15 AAVHSCs. Homology believes that its compelling preclinical data, scientific expertise, product development strategy, manufacturing capabilities and intellectual property position it as a leader in the development of genetic medicines.

## A12 Identification of a novel kinase target for myotonic dystrophy therapy

### David Brook

#### University of Nottingham, Nottingham, NG7 2RD, UK

##### **Correspondence:** David Brook

*Journal of Translational Medicine* 2018, **16(Suppl 2):**A12

Myotonic Dystrophy type 1 (DM1), is an RNA-based disease caused by a transcribed CTG-repeat expansion within the 3’ UTR of the DMPK gene. Mutant repeat expansion transcripts remain in the nucleus of patient’s cells, forming foci that contribute substantially to the pathophysiology of the condition. Previously we developed a high throughput screening assay based around this disease feature and identified kinase inhibitors that were successful in removing these nuclear foci. We have sought to identify the kinase target responsible. We utilised a high content imaging screen to assess a large, well-characterised kinase inhibitor library for compounds that reduce nuclear foci to identify their common targets. A combination of chemoproteomics and mass spectrometry revealed a novel kinase target in DM pathophysiology. This kinase was analysed by western blot analysis and by detailed expression analysis in patient derived cells and biopsy samples. A compound inhibitor of this kinase was tested in the HSALR mouse model to assess phenotypic effects. The novel kinase is elevated in DM1 cell lines and patient muscle biopsies. Inhibition leads to the dissolution of nuclear foci and degradation of repeat expansion transcripts. Inhibitor treatment in the HSALR mouse led to beneficial phenotypic effects on myotonia and improvement of key splice isoforms. Our methods validate the use of phenotypic screening in drug discovery and highlight a collection of molecules suitable for further development in the therapeutic effort against DM1.

## A13 A non-profit mechanism for moving proof-of-concept studies to clinical therapies for rare retinal disorders

### Scott Dorfman

#### Odylia Therapeutics, Boston, MA, USA

##### **Correspondence:** Scott Dorfman

*Journal of Translational Medicine* 2018, **16(Suppl 2):**A13

With now multiple modalities of gene therapy achieving regulatory approval, a growing body of clinical experience has supported commercial investment in gene therapy technologies. However, given the prohibitive costs of vector development, this investment has largely been limited to indications for which sufficiently large patient populations exist to support a financially viable model of commercialization. There exist many therapies for rare disorders that have robust pre-clinical proof-of-concept but due to ultra-rare prevalence lack a viable commercial model. Ultra-rare disease needs to establish new pre-clinical and clinical models that recognize the costs associated with bringing therapies to the clinic. Odylia Therapeutics, a non-profit gene therapy company, bridges the gap between researchers and drug developers by conducting preclinical and clinical development of gene therapies. Odylia hopes to usher in novel efficiencies and economies of scale in pre-clinical and clinical vector development. Odylia has designed a strategic plan that focuses on redefining a lean yet safe commercialization strategy, allowing therapies for rare disease to progress to the clinic more efficiently. Odylia precisely and iteratively determines how to increase efficiency and effectively move more therapies from the lab to the clinic. Odylia seeks to redefine lean clinical trials, including a new paradigm of a combined phase I/II/III clinical trial, which will increase patient access to emerging therapies. Odylia’s pre-clinical and clinical processes are made available for collaboration with for-profit development companies. Through these for-profit/non-profit collaborations, Odylia represents a new paradigm of drug development which will enable previously non-viable therapies to reach clinical viability.

